# Development of a prototype blood fractionation cartridge for plasma analysis by paper spray mass spectrometry

**DOI:** 10.1016/j.clinms.2016.12.002

**Published:** 2016-12-09

**Authors:** Brandon J. Bills, Nicholas E. Manicke

**Affiliations:** Department of Chemistry and Chemical Biology, Indiana University-Purdue University Indianapolis, Indianapolis, IN, United States

## Abstract

Drug monitoring of biofluids is often time consuming and prohibitively expensive. Analysis of dried blood spots offers advantages, such as reduced sample volume, but depends on extensive sample preparation and the presence of a trained lab technician. Paper spray mass spectrometry allows rapid analysis of small molecules from blood spots with minimal sample preparation, however, plasma is often the preferred matrix for bioanalysis. Plasma spots can be analyzed by paper spray MS, but a centrifugation step to isolate the plasma is required. We demonstrate here the development of a paper spray cartridge containing a plasma fractionation membrane to perform automatic on-cartridge plasma fractionation from whole blood samples. Three commercially available blood fractionation membranes were evaluated based on: 1) accuracy of drug concentration determination in plasma, and 2) extent of cell lysis and/or penetration. The accuracy of drug concentration determination was quantitatively determined using high performance liquid chromatography–mass spectrometry (HPLC–MS). While the fractionation membranes were capable of yielding plasma samples with low levels of cell lysis, the membranes did exhibit drug binding to varying degrees, as indicated by a decrease in the drug concentration relative to plasma obtained by centrifugation. Using the membrane exhibiting the lowest binding, we developed a composite paper spray cartridge incorporating the selected fractionation membrane. Quantitative analysis of the plasma samples by paper spray MS yielded results similar to those found with HPLC–MS, but without the need for offline extraction or chromatography.

## Highlights

• Development of a paper spray cartridge with integrated plasma fractionation.
• Three commercially available plasma separation membranes were evaluated.
• HPLC–MS/MS was performed on five drugs in plasma from membrane fractionation.
• Drug binding to fractionation membranes was found to be dependent on drug properties.
• A cartridge is presented with quantitative performance comparable to HPLC–MS.

## 1 Introduction

Monitoring biofluid drug concentration is important for a number of applications. Pharmaceuticals, for example, sometimes require that concentrations be maintained within patient-specific ranges to effect desired outcomes and, if possible, avoid toxicity. Since individuals metabolize drugs at variable rates [1] it would be useful to routinely monitor drug concentrations to ensure optimal efficacy. Current methods that analyze whole blood require time-consuming sample preparation. Whole blood also requires special handling and refrigeration during transit. Because of these factors, therapeutic drug monitoring is often prohibitively expensive. Many fields in which small molecules are being monitored, such as forensics and toxicology, are faced with a similar problem. To address this need, there exists a continual push to develop rapid and cost effective analytical techniques that require minimal sample handling and preparation.

In 2010, paper spray mass spectrometry (PS-MS) emerged as a facile technique requiring minimal sample preparation [2], [3]. PS-MS is an ambient ionization technique in which the sample is contained on a paper substrate. The paper is placed in front of the atmospheric pressure inlet of a mass spectrometer, and solvent is eluted through the sample, extracting the analytes [4]. A high voltage applied to the paper generates a plume of charged droplets, which produce a mass spectrum characteristic of (electrospray ionization) ESI [2]. PS-MS has been demonstrated as a capable method for the analysis of biofluids, such as blood and urine [2], [3], [5], [6]. Direct analysis of blood spots reduces sample preparation and minimizes the volume of sample required. Because of its simplicity, PS-MS has potential for point-of-care analysis [7], [8], [9]. Dried blood spots do not require the special handling or refrigeration of whole blood samples. Thus, even if a point-of-care option is unavailable, the sample could be shipped as a dried spot. In addition, analyte stability is generally enhanced in a dried blood matrix [10], [11]. PS-MS has been shown to be useful in the quantitation of a wide variety of pharmaceuticals including immunosuppressive drugs, such as tacrolimus and cyclosporine [12], [13], as well as illicit drugs [14], [15], [16].

The primary disadvantages of paper spray MS relative to HPLC–MS is a lower selectivity caused by a lack of chromatography, and a lower sensitivity caused by matrix effects [17]. These disadvantages can be partially ameliorated via ion mobility [18], on-cartridge pre-concentration via solid phase extraction (SPE) [19], solid-phase microextraction (SPME) in which a spray substrate is immersed in a large sample volume [20], [21], and alternative substrates that improve detection for particular analytes [22], [23], [24], [25], [26].

One limitation of dried blood spot analysis is that it is the analyte concentration in plasma, not whole-blood, that is often desired [27]. In general, the use of plasma, instead of blood, is more readily accepted in pharmacokinetic studies [28]. This is especially true for anti-psychotic drugs where drug plasma concentration correlates to blocked receptors and efficacy [29]. Another concern is inaccuracy caused by a variable hematocrit [30]. Centrifugation, the typical method of obtaining plasma from whole blood, adds another step in the analysis and also requires a dedicated piece of equipment. Several methods for obtaining plasma from whole blood, without resorting to centrifugation, have been reported and range from acoustics [31] to labyrinth-like mazes [32]. While effective, these methods are complex and may not be feasible for incorporation as part of a disposable collection device. Blood fractionation membranes offer another potential solution. A blood fractionation card has been described that is able to obtain plasma from capillary blood in a timely manner with negligible lysis [33]. Another recent study found that two different fraction membranes were capable of obtaining plasma from whole blood and yielding accurate and precise analytical results for the drug guanfacine [34]. Another technique for obtaining plasma for colorimetric assays employed agglutination of red blood cells using paper treated with antibodies, which allowed isolation of plasma from the agglutinated cells [35].

Paper spray can be used to analyze dried plasma spots. For example, a paper spray cartridge was developed with an integrated solid-phase extraction column to perform analyte pre-concentration for analysis of plasma samples [19]; however, this method required centrifugation. Here, we investigate the use of several plasma separation membranes for small molecule drug analysis using both HPLC- and paper spray MS/MS. Our goal is to develop an inexpensive cartridge for rapid, direct analysis of plasma obtained from a drop of whole blood. Using HPLC–MS/MS, three commercially available blood fractionation membranes were evaluated based on their ability to obtain hemolysis-free plasma without altering small molecule drug concentrations. The membranes were incorporated into a simple paper spray cartridge to enable direct plasma analysis after application of a drop of whole blood. Addition of agglutinating agents to the membranes was also investigated to determine if they could improve blood fractionation without interfering with drug detection.

## 2 Materials and methods

### 2.1 Materials

All drugs and their stable isotopic labels (SIL), except atenolol D7, were obtained from Cerilliant (Round Rock, TX). Atenolol D7 was obtained from CDN isotopes (Pointe-Claire, QC, Canada) as a powder (⩾98% purity). HPLC-grade methanol, acetonitrile, formic acid and acetic acid came from Fisher Scientific (Waltham, MA). Five individual donor human blood samples came from Innovative Research in K_2_EDTA-treated vials (Novi, MI). NoviPlex cards were obtained from Novilytic (West Lafayette, IN), while CytoSep and Vivid blood fractionation membranes were obtained from the Pall Corporation (Port Washington, NY). The holder used to contain the Vivid membrane was 3D printed using a Stratasys Objet 30 pro using VeroBlue photopolymer (Eden Prairie, MN). The paper spray cartridges and other sample holders were machined out of Delrin and HDPE plastic, respectively (McMaster-Carr) (Elmuhurst, IL) on a bench-top mini milling machine (Sherline – Vista, CA). Whatman grade 31 ET chromatography paper was used for the spray substrate and sample punches (GE Healthcare Life Sciences – Pittsburgh, PA). Human fibrinogen (50–70% protein), alum (aluminum potassium sulfate dodecahydrate ⩾98% purity), carbamazepine powder (⩾98% purity), and NaCl were obtained from Sigma-Aldrich (St. Louis, MO).

### 2.2 Plasma separation

**Fig. 1:**
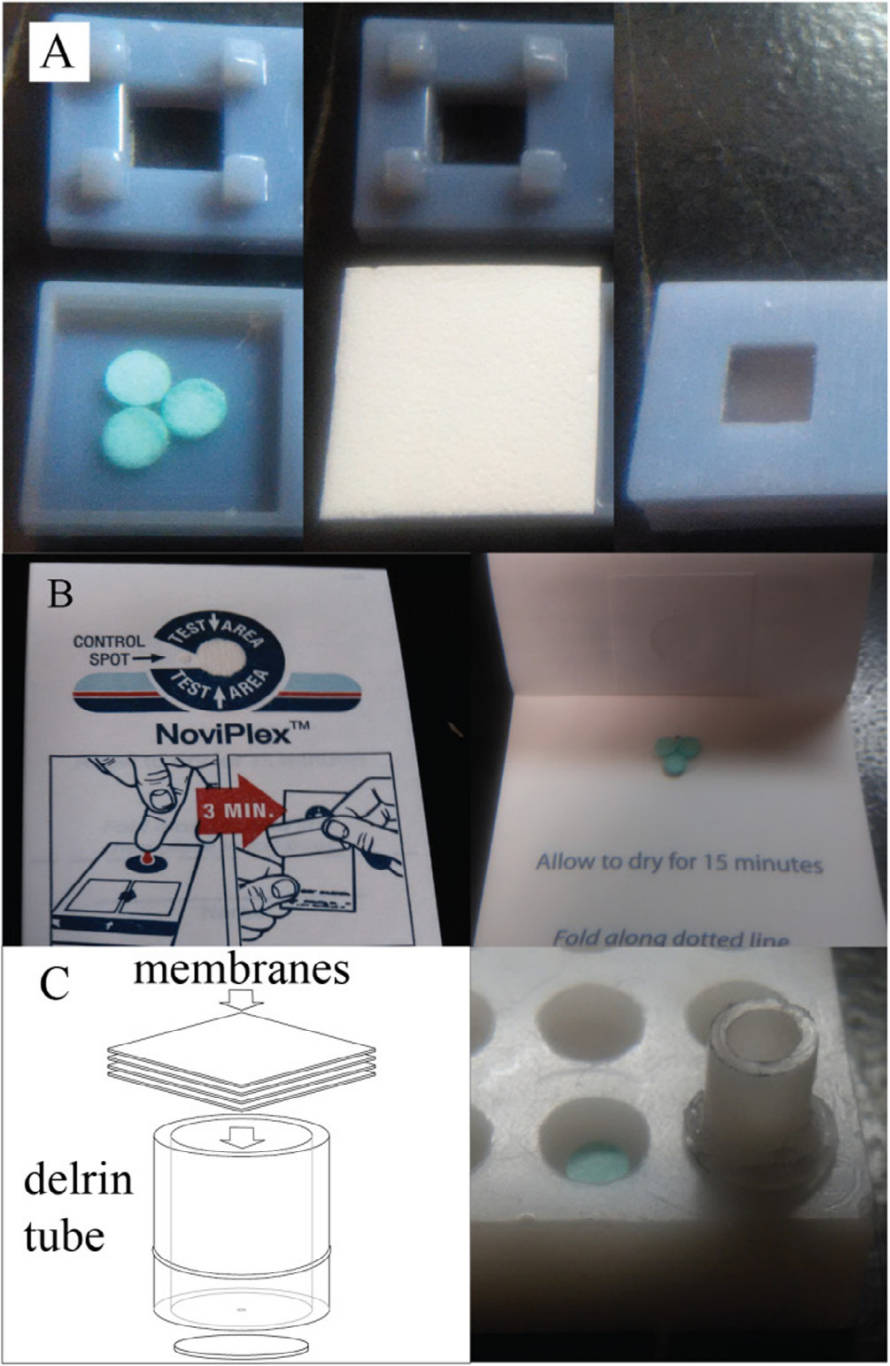
Example membrane configurations for plasma fractionation from whole blood. Sample punches are dyed green here for contrast. (A) Sample holder for the vivid membrane shown left to right: holder with the sample punches, 13 mm square of vivid membrane, and closed holder with membrane inside (B) Noviplex fractionation card with sample punches in place. (C) Diagram of delrin tube used to contain the 4 squares of CytoSep membrane (left) and delrin tube inside the holder with the CytoSep membranes already in the tube (right).

As shown in Fig. 1, each membrane required different experimental parameters to obtain extracted plasma. Sample punches consisted of 3 mm punches of Whatman 31ET filter paper. The Vivid GR membrane, shown in Fig. 1A, was prone to ripping, so a special holder was made to contain the punches and a 13 mm × 13 mm membrane. When the top piece of the holder was put into position, it forced the membrane into a bowl shape to hold a 40 μL blood sample while plasma wicked through to 3 sample punches over the course of 3 min. The NoviPlex plasma fractionation card, shown in Fig. 1B, was already designed to be a self-contained sampling device; the cards were used as designed, except the sample collection disc in the NoviPlex card was replaced with 3 of the same punches used in our other experiments. Fractionation with the NoviPlex card entailed spotting 30 μL of whole blood onto the top layer and allowing 3 min for plasma to wick through to the sample punch below. The CytoSep membrane was assembled inside a disposable tube by inserting four 5 mm × 5 mm squares of grade 1660 membrane, one at a time, as shown in Fig. 1C. Four stacked membranes were required to yield improved plasma fractionation. The disposable insert was a 9 mm length of Delrin tubing (3.175 mm inner diameter) with a piece of wax paper with a small hole in it glued over the bottom-end between the separation membrane and the collection disc. To obtain plasma, the insert containing the membranes and wax paper was placed in a tightly fitted HDPE holder with a sample punch beneath it, as shown in the picture on the right in Fig. 1C. Whole blood was applied to the top of the insert and 15 min was allowed for the plasma to wick through to the sample punch. For each sample, the wet mass of the sample punch was obtained using a microgram scale and the plasma mass was determined by subtracting the mass of the paper punch.

### 2.3 Sample preparation

The experimental samples for HPLC–MS/MS were prepared in five different lots of blood at two concentrations: 1.5 μg/mL and 0.3 μg/mL for each drug. Each blood sample was taken through the three different membrane fractionation methods in triplicate. Drug stock solutions were diluted to working solutions in 0.6 mg/mL aqueous saline before being spiked into whole blood to minimize solvent-based cell lysis. All spiked blood samples had a maximum methanol content of 1.5% (v/v). Samples were incubated at least 1 h at 37 °C prior to fractionation and were applied to the membrane fractionation apparatus while still warm. The plasma punches were dried for 1 h at room temperature in open air. Plasma control samples were prepared by centrifuging an aliquot of the blood samples at 1500 rpm for 30 min.

Calibrators and calibration verification standards were prepared in drug-free plasma pooled from 5 blood samples. The calibration curve was prepared by spiking the pooled plasma with 0.03, 0.1, 0.3, 1 and 3 μg/mL each of atenolol, carbamazepine, fentanyl, nortriptyline, and methadone. A calibration verification sample was prepared separately at 1 μg/mL. Calibrators and calibration verification samples were spotted on paper punches identical to those used for the fractionated samples. The paper punches contained 2.3 mg of the calibration samples, which corresponded to the average wet mass of plasma obtained using the three different plasma fractionation membranes. An analytical balance was used to measure the mass of plasma for both the calibrators and the membrane fractionated samples; these masses were required to normalize the plasma volume of the HPLC–MS calibrators (see next section). Calibrators were run in duplicate, and a calibration verification standard was run every 10 samples.

To determine if PS-MS/MS could be used to obtain quantitative results similar to those of HPLC–MS/MS, plasma extracted using the fibrinogen-treated membranes was compared to centrifuged plasma samples. The curve and samples were prepared as described for the HPLC–MS/MS experiments. The samples came from a single lot of blood with 6 replicates for the membrane separated samples and 5 replicates for the centrifuged control samples.

### 2.4 HPLC–MS/MS

Dried sample punches were transferred to a 500 μL centrifuge tube containing 100 μL of extraction solvent (15% methanol by volume in water containing 0.1 μg/mL each of atenolol D7, carbamazepine D10, fentanyl D5, nortriptyline D3 and methadone D3). Samples were vortexed for 30 min. The sample punches were then removed and the solutions were centrifuged at 3000 rpm for 5 min. From each sample, an 80 μL aliquot was transferred to an HPLC vial for analysis. Analysis was carried out using an Agilent G2226A nanopump and G1379A degasser with an Agilent G1329A autosampler (Agilent Technologies, Santa Clara, CA) equipped with a hypersil gold C18 column (Thermo Scientific, San Jose, CA) coupled to a Thermo Scientific LTQ XL mass spectrometer. The sample volume injected was 15 μL. The HPLC separation was a gradient elution (Solvent A was water with 0.01% formic acid, solvent B was methanol with 0.01% formic acid) at 200 mL/min. The gradient was as follows: 15% Solvent B (2 min), followed by a linear increase to 80% Solvent B over 2 min, then to 95% over 1.2 min. Solvent B was held at 95% methanol for 4.5 min. The column was re-equilibrated between runs for 5 min with 400 μL/min of 15% Solvent B. MS was conducted in positive ion mode using electrospray ionization (ESI). The sheath gas was set to 20 while the auxiliary gas was set to 5. The spray voltage was 4.5 kV. The instrument was operated in MS/MS mode. The area under the curve was determined for a unique fragment of the [M+H]^+^ ion for each analyte and ratios were determined by dividing the peak area for the analyte by the peak area for the SIL. The slope and y-intercept of the curve for each drug was found using 1/x weighted least squares [36]. Analysis of all samples obtained from the three different membranes was performed in one continuous run based off a single curve. Because each of the three membrane types yielded a different mass of plasma, final concentrations obtained from the calibration curve were normalized based on the average mass of plasma obtained for the each membrane type using Eq. (1):

(1) $$C_{f}=C_{i}\left(\frac{M_{\mathrm{calibration}}}{M_{\mathrm{sample}}}\right)$$

where *C_f_* is the final determined concentration, *C_i_* is the concentration calculated without normalizing the mass of the plasma calibrators, *M_calibration_* is the average mass of plasma spotted for the calibrators, and *M_sample_* is the average mass of plasma obtained for a particular plasma fractionation method. *M_calibration_* was 2.3 mg. *M_sample_* values were 2.2 mg, 2.8 mg, and 1.5 mg for the cytosep, vivid, and the noviplex membranes, respectively.

Limits of detection (LOD) for both paper spray and HPLC–MS/MS were estimated using the formula LOD = (3 * *s_B_*)/*m*, where *s_B_* is the standard error of the intercept and *m* is the slope of the calibration line.

### 2.5 Paper spray analysis

**Fig. 2:**
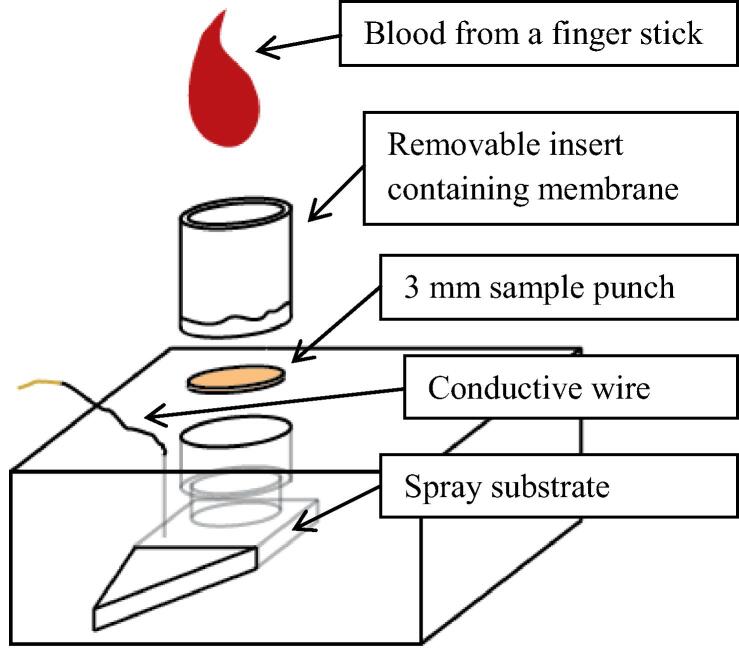
Schematic for paper spray cartridge with removable blood fractionation insert. The plastic is Delrin and the sample punch and spray substrate are Whatman grade 31 ET filter paper.

Plasma fractionation and PS-MS was carried out on a custom-built spray cartridge shown in Fig. 2. The removable insert was used for trials involving the CytoSep membrane as described above. The blood sample was applied to the removable tube, which contained the fractionation membranes in contact with a sample punch below the tube. When the punch was saturated after about 15 min, the tube insert was removed and discarded. An aliquot of 0.5 μg/mL SIL in methanol, with a volume equivalent to the average mass of extracted plasma, was spotted onto the dried plasma punch. After drying, the paper disc containing the plasma sample was pushed down onto the spray substrate.

Analysis was performed by positioning the cartridge in front of an LTQ XL mass spectrometer with the paper tip 2.5 mm away from the MS inlet. A 30 μL aliquot of spray solvent was pipetted on top of the sample disc, where it wicked through the sample and onto the spray substrate. The spray solvent was 95:5:0.01 methanol:water:acetic acid, except for cotinine samples, for which 90:10:0.01 acetonitrile:water:acetic acid was used. A voltage of 4.5 kV was applied for 60 s, via the wire contacting the spray substrate, to initiate the plume of ions. Analysis was carried out in MS/MS mode using the base peak fragment of the [M+H]^+^ for quantitation. For experimental purposes, one spray cartridge was re-used for each analysis and sample preparation was conducted in a block of plastic with holes as shown in Fig. 1C. Between runs, the cartridge was cleaned with methanol and the spray substrate was replaced.

### 2.6 Determining hematocrit and membrane induced hemolysis

The hematocrit was determined for each of the five lots of blood by filling a 25 mm segment of a borosilicate glass capillary (0.86 mm inner diameter Sutter Instrument, Novato, CA) with blood followed by sealing the tube with paraffin wax and centrifuging at 1500 rpm for 30 min. Hematocrit was calculated by measuring the packed cell volume relative to the total sample volume. The extent of red blood cell lysis and penetration in the membrane fractionated plasma samples was determined by measuring the absorbance of the sample from the extracts used for HPLC-MS/MS at 413 nm using a Thermo Scientific NanoDrop 2000 spectrophotometer. A calibration curve was created by mixing whole blood (a mix of equal parts of all 5 lots of blood) with centrifuged plasma to make calibrants ranging from 0.45% to 9% (v/v) cellular material in plasma. Blood cell content calibrators were dried and extracted as described for the experimental samples.

### 2.7 Membrane treatment to improve plasma separation

Fibrinogen concentrations ranging from 20 to 1000 μg/mL were tested for separation efficiency and potential drug binding on the CytoSep membrane. Fibrinogen solutions were spotted onto each membrane in the stack in four aliquots of 5 μL allowing roughly an hour between aliquots for drying at room temperature. The non-protein based agglutinating agent, alum, was also tested by treating different arrangements of 3 CytoSep membranes treated with varying combinations of alum solutions ranging in concentration from 20 to 60 mg/mL. In the final configuration only the top membrane was treated with 5 μL of 40 mg/mL alum solution.

## 3 Results and discussion

### 3.1 Hematocrit, plasma mass and lysis/cell penetration

Three commercially available plasma separation membranes were evaluated for their ability to obtain reproducible amounts of red blood cell-free plasma (Table 1). Five lots of human blood, with hematocrits ranging from 45 to 54%, were evaluated. The average mass of plasma obtained for each membrane was calculated from 30 samples (6 replicates from each lot of blood) and the % volume of red blood cells was determined based on 5 samples (1 replicate from each lot). Both the Vivid GR and Noviplex card showed good plasma fractionation with a total red blood cell content below 1% (v/v). The Cytosep grade 1660 membrane stacked 4 high showed noticeably reduced separation, most likely due to the lateral flow characteristic of the membrane.

**Table 1:** Average and 95% confidence interval for the wet plasma mass obtained for each membrane (N = 30 across 5 blood lots) and average and 95% confidence interval red blood cell content in the plasma (N = 5 for each method except Vivid, which had 4 due to a small amount of leaked blood in the holder).

Fractionation membrane	Mass of plasma obtained (mg)	Red blood cell % (v/v)
CytoSep grade 1660	2.2 ± 0.4	6 ± 2
Vivid GR	2.82 ± 0.06	0.22 ± 0.09
Noviplex	1.53 ± 0.04	0.8 ± 0.2

### 3.2 Assessment of drug binding

Drug binding was evaluated by measuring the drug concentration of the plasma obtained by membrane filtration and determining the % decrease relative to plasma obtained by centrifugation (Table 2). Calibrators and calibration verification samples were prepared directly in plasma to ensure a known plasma concentration. The experimental samples were prepared in whole blood, as described in the Materials and Methods section, to allow the drugs to distribute between the blood cells and the plasma. Drug quantitation was performed using HPLC–MS/MS. The raw concentration data can be found in Supplementary Table 1.

**Table 2:** The average % decrease in drug concentration relative to the concentration found in centrifuged plasma for three commercially available membranes at two concentrations.

Drug	Concentration	CytoSep	Vivid	Noviplex
Atenolol	0.3 μg/mL	−13%	−36%	−33%
	1.5 μg/mL	0%	−36%	−30%

Carbamazepine	0.3 μg/mL	−17%	−65%	−51%
	1.5 μg/mL	−8%	−71%	−62%

Fentanyl	0.3 μg/mL	−31%	−76%	−31%
	1.5 μg/mL	−28%	−79%	−31%

Methadone	0.3 μg/mL	−55%	−66%	−50%
	1.5 μg/mL	−42%	−45%	−36%

Nortriptyline	0.3 μg/mL	−78%	−57%	−29%
	1.5 μg/mL	−76%	−64%	−30%

Both the Vivid GR and the NoviPlex membranes caused a measurable and significant decrease in the concentration for all five drugs. The CytoSep membrane showed a noticeable decrease for three drugs, but not for atenolol and carbamazepine. The presumed cause of the decrease in drug concentration in membrane-fractionated plasma is drug adsorption to the membrane. For example, the Vivid membrane is polysulfone–based, and it has been shown in several studies that polysulfone-based membranes can cause a decrease in drug concentration in the blood when used for dialysis [37], [38].

### 3.3 Membrane treatment to improve plasma separation

The CytoSep membrane showed the lowest amount of drug binding, but also resulted in the highest level of red blood cell content in the collected plasma sample. We investigated methods for improving the plasma fractionation efficiency from this membrane. Previous work showed that agglutinating cells allowed for plasma to be separated from blood cells in paper-based devices [35]. However, this method employed anti-A and anti-B antibodies to effect the agglutination, and thus its application is limited to blood types which express these antigens, and would not be effective in causing agglutination of type O blood [35]. We chose alternative agglutination agents: fibrinogen, a key protein in the coagulation cascade [39], and alum [40]. Fibrinogen and alum were spotted directly onto the membranes as described in the methods section above and allowed to dry before assembly of the insert.

Fibrinogen concentrations ranging from 20 to 1000 μg/mL were tested. Fibrinogen concentrations of 75 μg/mL or greater were deemed unacceptable, because they showed measurable drug binding for the initial test set of drugs. The 50 μg/mL fibrinogen-treated membranes showed improved cell lysis/penetration of approximately 3% compared to approximately 6% for the untreated membrane. Concentrations less than 50 μg/mL showed minimal improvement. Hence, the 50 μg/mL fibrinogen-treated membrane was selected for further evaluation.

Similarly, different concentrations of alum were evaluated for their effects on separation efficiency. However, it was found that the amount of alum required to improve the plasma separation also yielded irreproducible amounts of plasma on the collection disc. A lower concentration (i.e., 40 mg/mL instead of 60 mg/mL) of alum was chosen for further testing, as described in the methods section, even though the separation efficiency was similar to that of the untreated membrane. This testing was performed to evaluate if a non-protein based agglutination agent would have a noticeable impact on the binding properties of the drugs.

### 3.4 Evaluation of errors from drug binding and cell lysis

A selection of drugs with varying logP and K_B/P_ values were tested to better understand the binding characteristics of the treated and untreated CytoSep membrane, as well as the errors caused by blood cell lysis. Plasma samples were applied to the membrane fractionation device in addition to whole blood samples to evaluate the hypothesis that plasma samples could be used to evaluate membrane drug binding. This would be advantageous because using plasma samples to evaluate drug binding removes the complicating effects of red blood cell lysis/penetration. The results are shown in Table 3.

**Table 3:** Deviation in drug concentration in plasma obtained using CytoSep grade 1660 membrane (both untreated and treated with alum or fibrinogen) relative to plasma obtained via centrifuge. Drug concentrations ranged from 250 to 1000 ng/mL. NS indicates that the difference between the results for the membrane-fractionated and centrifuged plasma were not statistically significant (p > 0.05).

Drug	logP	K_B/P_	Deviation for plasma applied to untreated membrane	Deviation for blood applied to untreated membrane	Deviation for blood applied to fibrinogen membrane	Deviation for blood applied alum membrane
Selegiline	3.08	1.7	−5% NS	−26%	−43%	−21%
Chlorpheniramine	3.74	1.34	−5% NS	−31%	−46%	−18%
Atenolol	0.57	1.07	8% NS	−2% NS	−8% NS	−4% NS
Carbamazepine	2.1	1.06	13% NS	5% NS	−3% NS	−10% NS
Fentanyl	4.02	0.97	−3% NS	−31%	−24%	−31%
Cotinine	0.39	0.88	−4% NS	−15%	−19%	−0% NS
Alprazolam	2.23	0.78	5% NS	−14%	−11%	−37%
Methadone	4.14	0.75	−10% NS	−51%	−45%	−13%
Diazepam	2.63	0.58	15% NS	−4% NS	17%	−6% NS

None of the nine drugs tested exhibited significant binding when plasma samples were applied to the membrane. However, when whole blood was applied to the membrane, rather than plasma, six of the drugs showed a measurable decrease in analyte concentration. The two most probable sources of error are cell lysis/penetration or increased membrane-binding from blood compared to plasma. If cell lysis/penetration was the principal source of error in blood, then the error after passing through the membrane should be negative for drugs with a K_B/P_ < 1, positive for drugs with a K_B/P_ > 1, and minimal for drugs with a K_B/P_ close to 1. The drug concentration deviation did not correlate with the K_B/P_, however. For example, selegiline and chlorpheniramine have a K_B/P_ well above 1, but showed a significant decrease in blood concentration after passing through the membrane. Methadone had a negative error, but it was much greater in magnitude than would be expected based on the measured levels of cell lysis/penetration and its K_B/P_. We hypothesize that the error is caused rather by higher levels of drug binding to the membrane in blood samples compared to plasma. One possible reason for the higher levels of membrane drug binding in blood samples compared to plasma samples is the slower passage of blood samples through the membrane. Plasma samples pass through the membrane considerably faster due to their lower viscosity and because the membrane is not clogged by blood cells. The decreased extraction time (roughly 5 times shorter than with whole blood) could lead to less binding due to the shorter interaction time with the membrane. Another possibility is that the cellular material in the whole blood samples contributes to drug binding as it accumulates on the membrane. Either way, plasma is not a suitable matrix for evaluating drug binding by the fractionation membranes.

Table 3 also shows the logP (octanol-water) for each analyte. All of the drugs with a logP of 3 or greater exhibited negative deviations of 20% or more against the untreated membrane in whole blood samples. All the drugs with a logP below 3, on the other hand, deviated by 15% or less. This suggests that drugs with a K_B/P_ close to 1 and a logP below 3 are the least likely to be biased by membrane fractionation.

Membranes treated with agglutinated agents were also evaluated. Whole blood samples were applied to the treated membranes, and the concentration in the fractionated plasma was compared to centrifuged plasma from the same source. Although fibrinogen significantly decreased the amount of cell lysis/penetration, it did not improve the analyte error. In two cases, selegiline and chlorpheniramine, larger negative error was observed. This could be due to the lower amount of cell lysis/penetration in the fibrinogen-treated membrane; for drugs with K_B/P_ values > 1, the error from cell lysis/penetration should partially offset the error from drug binding. For the alum-treated membranes, the amount of cell lysis/penetration was not significantly different than the untreated membrane. There was a measurable decrease in analyte binding relative to the untreated membrane for two of the drugs (cotinine and methadone), but one of the drugs (alprazolam) showed increased binding.

### 3.5 Quantitative analysis by paper spray MS/MS

**Fig. 3:**
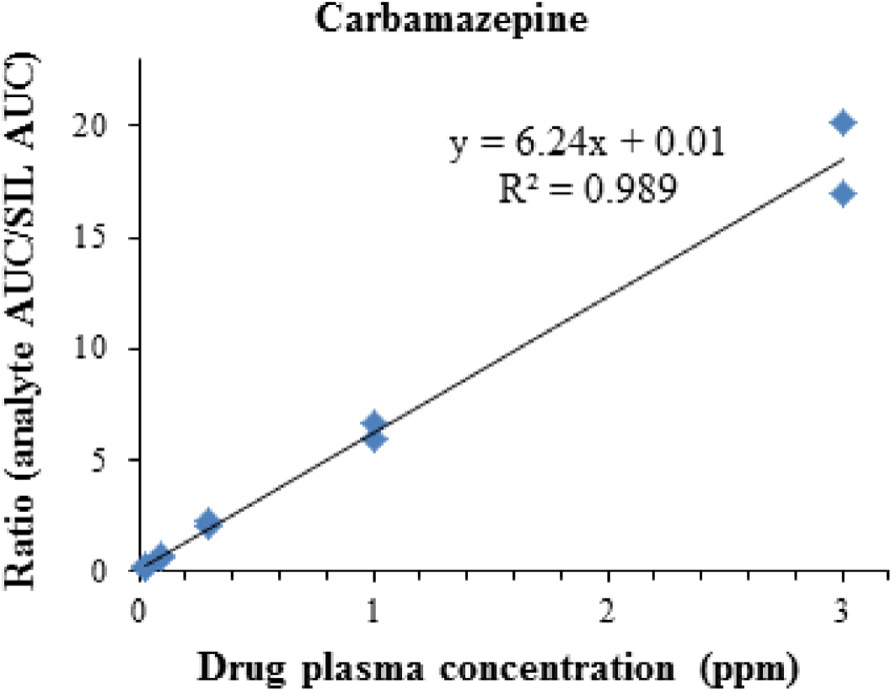
Paper spray MS/MS calibration curve for carbamazepine. X-axis is the concentration of drug spiked into the plasma. y-axis is a ratio of the instrument response to the unlabeled drug to the isotopically labeled internal standard.

We built a prototype paper spray cartridge incorporating a plasma fractionation membrane (Fig. 2). A drop of blood applied to the cartridge yields a plasma spot that can be analyzed directly from the cartridge via paper spray MS without any additional sample preparation. To evaluate the ability of the method to quantify plasma concentrations, blood samples containing atenolol and carbamazepine at concentrations of 0.3 and 1.5 μg/mL were prepared. The plasma was then fractionated using the fibrinogen-treated membrane inserts and compared to centrifuged plasma from the same blood sample. An example paper spray calibration curve for carbamazepine is shown in Fig. 3. The results for the calibration curves, the limits of detection, and the deviation obtained for the membrane fractionated plasma samples, as compared to the centrifuged plasma control samples, are shown in Table 4. HPLC–MS/MS results for the same experiment are also shown in Table 4. The raw concentration data, with standard deviations for both HPLC–MS and PS-MS, can be found in Supplementary Tables 2 and 3, respectively. Comparing plasma obtained by centrifugation to the on-cartridge membrane fractionation, the determined atenolol and carbamazepine concentrations, as well as the limits of detection, were not significantly different. The use of HPLC–MS/MS rather than PS-MS/MS yielded similar results when comparing membrane fractionation and centrifuged plasma samples as shown in Table 4 and Supplementary Table 3. The PS-MS results, however, were obtained without an extraction step (30–35 min) or a HPLC run (around 14 min per sample). This significantly reduced the amount of time and effort required to obtain the result.

**Table 4:** Calibration curve R^2^, LOD, and the deviation of the plasma samples obtained by membrane-fractionation compared to centrifugation. Membrane-fractionated plasma was compared to centrifuged plasma at two levels (0.3 and 1.5 μg/mL of atenolol or carbamazepine). Results obtained using both HPLC–MS/MS and paper spray-MS/MS for the same experiment are shown for comparison. NS indicates that the difference between the membrane fractionated and centrifuged plasma samplers were not statistically significant (p > 0.05).

	Atenolol		Carbamazepine	
	PS-MS/MS	HPLC–MS/MS	PS-MS/MS	HPLC–MS/MS
Calibration curve R^2^	0.983	0.982	0.989	0.995
LOD from plasma (μg/mL)	0.04	0.03	0.03	0.02
Deviation between membrane fractionated and centrifuged plasma at 0.3 μg/mL	10% (NS)	−2% (NS)	−4% (NS)	−9% (NS)
Deviation between membrane fractionated and centrifuged plasma at 1.5 μg/mL	−9% (NS)	−0.4% (NS)	−10% (NS)	−8% (NS)

Ideally, the paper spray cartridge would be able to deliver a reproducible volume of plasma from poorly controlled blood sample volumes. We applied volumes of blood ranging from 30 to 50 μL to the treated membranes to determine if they yielded similar masses of plasma. Over a range of 20 μL, the volume of blood had no effect on the extracted volume of plasma (Supplementary Table 4).

## 4 Conclusion

Three different membranes were evaluated for lysis and drug binding in order to develop a disposable paper spray MS cartridge to analyze a dried plasma sample obtained from a drop of whole blood. The membranes were made from a variety of materials ranging from polymers to natural and synthetic fibers. Two of the three membranes, the Vivid polysulfone membrane and the NoviPlex plasma fractionation card, exhibited noticeable amounts of drug binding for each of the five drugs tested. The third membrane, CytoSep, exhibited no significant binding for two of the drugs, but did exhibit drug binding for others. While the CytoSep membrane had the most favorable drug binding characteristics, it showed the poorest fractionation efficiency, as measured by the red blood cell content in the fractionated plasma. Overall, this membrane could be used in quantitative analysis of drugs with a logP less than three and a K_B/P_ near unity, but is not a universal solution.

This work was done with the intent of developing a simple method for analyzing plasma from whole blood using paper spray mass spectrometry. We have demonstrated a proof-of-concept design for a simple paper spray cartridge to perform direct plasma analysis from a drop of blood. Using this cartridge, plasma sample could be obtained from an uncontrolled volume of whole blood, and direct analysis could then be performed on the plasma sample using paper spray MS without separate extraction or chromatography steps. Quantitative analysis using the paper spray MS cartridge was found to be comparable to extraction of dried plasma spots followed by HPLC–MS/MS. However, unbiased results are only obtainable for membrane-fractionated plasma for a subset of drugs due to the potential for drug binding and cell lysis. Further investigation is required to identify membrane materials that are capable of sufficient plasma fractionation efficiency, while minimizing non-specific drug binding.

## References

[1] Lu A.Y.H. (1998). Drug-metabolism research challenges in the new millennium individual variability in drug therapy and drug safety. Drug Metab. Dispos..

[2] Liu J. (2010). Development, characterization, and application of paper spray ionization. Anal. Chem. (Washington, DC, U. S.).

[3] Wang H. (2010). Paper spray for direct analysis of complex mixtures using mass spectrometry. Angew. Chem. Int. Ed..

[4] Manicke N.E., Bills B.J., Zhang C. (2016). Analysis of biofluids by paper spray MS: advances and challenges. Bioanalysis.

[5] Manicke N.E. (2011). Assessment of paper spray ionization for quantitation of pharmaceuticals in blood spots. Int. J. Mass Spectrom..

[6] Manicke N.E. (2011). Quantitative analysis of therapeutic drugs in dried blood spot samples by paper spray mass spectrometry: an avenue to therapeutic drug monitoring. J. Am. Soc. Mass Spectrom..

[7] Shen L. (2013). High throughput paper spray mass spectrometry analysis. Clin. Chim. Acta.

[8] Li L. (2014). Mini 12, Miniature mass spectrometer for clinical and other applications-introduction and characterization. Anal. Chem..

[9] Espy R.D. (2012). Rapid analysis of whole blood by paper spray mass spectrometry for point-of-care therapeutic drug monitoring. Analyst (Cambridge, U. K.).

[10] Spooner N., Lad R., Barfield M. (2009). Dried blood spots as a sample collection technique for the determination of pharmacokinetics in clinical studies: considerations for the validation of a quantitative bioanalytical method. Anal. Chem. (Washington, DC, U. S.).

[11] Waterman K.C., Adami R.C. (2005). Accelerated aging: prediction of chemical stability of pharmaceuticals. Int. J. Pharm..

[12] Shi R.-Z. (2016). Rapid measurement of cyclosporine and sirolimus in whole blood by paper spray-tandem mass spectrometry. Clin. Chem..

[13] Shi R.-Z. (2015). Rapid measurement of tacrolimus in whole blood by paper spray-tandem mass spectrometry (PS-MS/MS). Clin. Chim. Acta.

[14] Su Y. (2013). Quantitative paper spray mass spectrometry analysis of drugs of abuse. Analyst.

[15] Li M. (2014). Rapid, in situ detection of cocaine residues based on paper spray ionization coupled with ion mobility spectrometry. Analyst.

[16] Espy R.D. (2014). Paper spray and extraction spray mass spectrometry for the direct and simultaneous quantification of eight drugs of abuse in whole blood. Anal. Chem..

[17] Vega C. (2016). Ionization suppression and recovery in direct biofluid analysis using paper spray mass spectrometry. J. Am. Soc. Mass Spectrom..

[18] Manicke N.E., Belford M. (2015). Separation of opiate isomers using electrospray ionization and paper spray coupled to high-field asymmetric waveform ion mobility spectrometry. J. Am. Soc. Mass Spectrom..

[19] Zhang C., Manicke N.E. (2015). Development of a paper spray mass spectrometry cartridge with integrated solid phase extraction for bioanalysis. Anal. Chem..

[20] Gomez-Rios G.A., Pawliszyn J. (2014). Development of coated blade spray ionization mass spectrometry for the quantitation of target analytes present in complex matrices. Angew. Chem. Int. Ed..

[21] Deng J. (2014). Coupling solid-phase microextraction with ambient mass spectrometry using surface coated wooden-tip probe for rapid analysis of ultra trace perfluorinated compounds in complex samples. Anal. Chem..

[22] Zhang M. (2015). Membrane electrospray ionization for direct ultrasensitive biomarker quantitation in biofluids using mass spectrometry. Anal. Chem..

[23] Damon D.E. (2016). Direct biofluid analysis using hydrophobic paper spray mass spectrometry. Anal. Chem..

[24] Zhang Z. (2012). Silica coated paper substrate for paper-spray analysis of therapeutic drugs in dried blood spots. Anal. Chem..

[25] Wong M.Y.-M. (2014). Negative electrospray ionization on porous supporting tips for mass spectrometric analysis: electrostatic charging effect on detection sensitivity and its application to explosive detection. Analyst.

[26] Wong M.Y.-M. (2013). Electrospray ionization on porous spraying tips for direct sample analysis by mass spectrometry: enhanced detection sensitivity and selectivity using hydrophobic/hydrophilic materials as spraying tips. Rapid Commun. Mass Spectrom..

[27] Uges D.R. (1988). Plasma or serum in therapeutic drug monitoring and clinical toxicology. Pharm. Weekbl. Sci..

[28] Emmons G., Rowland M. (2010). Pharmacokinetic considerations as to when to use dried blood spot sampling. Bioanalysis.

[29] Hiemke C. (2011). AGNP consensus guidelines for therapeutic drug monitoring in psychiatry: update 2011. Pharmacopsychiatry.

[30] Timmerman P. (2013). Update of the EBF recommendation for the use of DBS in regulated bioanalysis integrating the conclusions from the EBF DBS-microsampling consortium. Bioanalysis.

[31] A. Doria, M. Patel, A.P. Lee, Rapid blood plasma separation with air-liquid cavity acoustic transducers, 2012, Chemical and Biological Microsystems Society.

[32] H. Tsutsui, H. Miyagawa, M. Yano, Blood plasma separator using micro pillars arranged like a labyrinth, 2014, Chemical and Biological Microsystems Society.

[33] Kim J.-H. (2013). Simple, miniaturized blood plasma extraction method. Anal. Chem. (Washington, DC, U. S.).

[34] Sturm R. (2015). Novel membrane devices and their potential utility in blood sample collection prior to analysis of dried plasma spots. Bioanalysis.

[35] Yang X. (2012). Integrated separation of blood plasma from whole blood for microfluidic paper-based analytical devices. Lab Chip.

[36] Almeida A.M., Castel-Branco M.M., Falcao A.C. (2002). Linear regression for calibration lines revisited: weighting schemes for bioanalytical methods. J. Chromatogr. B: Anal. Technol. Biomed. Life Sci..

[37] Tsuruoka S. (2004). Adsorption of oxacalcitriol by polysulphone haemodialyser in patients with secondary hyperparathyroidism. Br. J. Clin. Pharmacol..

[38] Petejova N. (2012). Vancomycin removal during low-flux and high-flux extended daily hemodialysis in critically ill septic patients. Biomed. Pap..

[39] Jan K.M., Chien S. (1973). Role of the electrostatic repulsive force in red cell interactions. Bibl. Anat..

[40] Espy R.D. (2012). Rapid analysis of whole blood by paper spray mass spectrometry for point-of-care therapeutic drug monitoring. Analyst.

