# Supplementary material for: Development of a prototype blood fractionation cartridge for plasma analysis by paper spray mass spectrometry
Source: Clin Mass Spectrom. 2016 Dec 9;2:18–24. doi: 10.1016/j.clinms.2016.12.002 (PMC11322750; doi:10.1016/j.clinms.2016.12.002)
Supplement: Supplementary data 1 [file mmc1.docx]

**Supplemental Information**

On-cartridge Blood Fractionation for Dried Plasma Analysis by Paper Spray Mass Spectrometry

Brandon J. Bills and Nicholas E. Manicke*

Department of Chemistry and Chemical Biology. Indiana University-Purdue University Indianapolis. Indianapolis, IN.

*nmanicke@iupui.edu

Supplementary table 1

Averaged plasma concentrations in µg/mL from whole blood at two concentrations (0.3 and 1.5 µg/mL). Plasma was separated using 4 grade 1660 CytoSep lateral flow membranes, Vivid GR asymmetric polysulphone membrane, Noviplex plasma prep card and centrifuge. Three samples from five different lots (N=15) were prepared for each concentration/separation method combination and analyzed using HPLC-MS/MS.

|  | CytoSep  0.3 µg/mL | CytoSep  1.5 µg/mL | Vivid GR  0.3 µg/mL | Vivid GR  1.5 µg/mL | Noviplex  0.3 µg/mL | Noviplex  1.5 µg/mL | Centrifuge  0.3 µg/mL | Centrifuge  1.5 µg/mL |
| --- | --- | --- | --- | --- | --- | --- | --- | --- |
| Atenolol | 0.32 | 1.665 | 0.237 | 1.075 | 0.247 | 1.171 | 0.368 | 1.673 |
| Carbamazepine | 0.265 | 1.414 | 0.112 | 0.439 | 0.157 | 0.586 | 0.32 | 1.532 |
| Fentanyl | 0.239 | 1.28 | 0.082 | 0.369 | 0.24 | 1.217 | 0.347 | 1.767 |
| Methadone | 0.211 | 1.043 | 0.159 | 0.988 | 0.234 | 1.151 | 0.469 | 1.788 |
| Nortriptyline | 0.063 | 0.364 | 0.126 | 0.543 | 0.205 | 1.058 | 0.29 | 1.506 |

Supplementary table 2

Averaged plasma concentrations with standard deviation in µg/mL from whole blood at two concentrations (0.3 and 1.5 µg/mL) using HPLC mass spectrometry. Plasma was separated using 4 grade 1660 CytoSep lateral flow membranes and centrifuge. N=15 for each sample.

|  | Cytosep  0.3 µg/mL | Cytosep  1.5 µg/mL | Centrifuge  0.3 µg/mL | Centrifuge  1.5 µg/mL |
| --- | --- | --- | --- | --- |
| Atenolol | 0.32±0.06 | 1.7±0.2 | 0.33±0.03 | 1.7±0.2 |
| Carbamazepine | 0.26±0.06 | 1.4±0.2 | 0.29±0.03 | 1.5±0.2 |

Supplementary table 3

Averaged plasma concentrations with standard deviation in µg/mL from whole blood at two concentrations (0.3 and 1.5 µg/mL) using paper spray mass spectrometry. Plasma was separated using 4 grade 1660 CytoSep lateral flow membranes and centrifuge. N=7 for the plasma separated using CytoSep membranes and N= 5 for the centrifuged samples.

|  | Cytosep  0.3 µg/mL | Cytosep  1.5 µg/mL | Centrifuge  0.3 µg/mL | Centrifuge  1.5 µg/mL |
| --- | --- | --- | --- | --- |
| Atenolol | 0.24±0.02 | 1.6±0.3 | 0.22±0.02 | 1.7±0.1 |
| Carbamazepine | 0.23±0.02 | 1.5±0.3 | 0.24±0.03 | 1.6±0.3 |

Supplementary table 4

The 95% confidence intervals for mass of plasma extracted from whole blood using fibrinogen treated CytoSep membranes from different volumes of whole blood (N=5).

| Volume of whole blood | Volume of plasma extracted (mg) |
| --- | --- |
| 30 µL | 2.7 ± 0.2 |
| 40 µL | 2.7 ± 0.2 |
| 50 µL | 2.7 ± 0.5 |
